# A novel 3-hydroxy-3-methylglutaryl-coenzyme A reductase *(HMGCR)* splice variant with an alternative exon 1 potentially encoding an extended N-terminus

**DOI:** 10.1186/1471-2199-13-29

**Published:** 2012-09-18

**Authors:** Camilla Stormo, Marianne K Kringen, Runa M Grimholt, Jens P Berg, Armin P Piehler

**Affiliations:** 1 Department of Medical Biochemistry, University of Oslo and Oslo University Hospital, Ullevål, P.O box 4956, Nydalen, Oslo, 0424, Norway; 2 Department of Pharmacology, Oslo University Hospital, Ullevål, P.O box 4956, Nydalen, Oslo, 0424, Norway; 3 Institute of Clinical Medicine, Faculty of Medicine, University of Oslo, P.O box 1171, Blindern, Oslo, 0318, Norway; 4 Fürst Medical Laboratory, Søren Bullsvei 25, Oslo, NO-1051, Norway

**Keywords:** 3-hydroxy-3-methylglutaryl-coenzyme A reductase, HMG-CoA, Transcription, Alternative splicing, Statin, Cholesterol

## Abstract

**Background:**

The major rate-limiting enzyme for de novo cholesterol synthesis is 3-hydroxy-3-methylglutaryl-coenzyme A reductase (HMGCR). HMGCR is sterically inhibited by statins, the most commonly prescribed drugs for the prevention of cardiovascular events. Alternative splicing of *HMGCR* has been implicated in the control of cholesterol homeostasis. The aim of this study was to identify novel alternatively spliced variants of *HMGCR* with potential physiological importance.

**Results:**

Bioinformatic analyses predicted three novel *HMGCR* transcripts containing an alternative exon 1 *(HMGCR-1b, -1c, -1d)* compared with the canonical transcript *(HMGCR-1a)*. The open reading frame of the *HMGCR-1b* transcript potentially encodes 20 additional amino acids at the N-terminus, compared with *HMGCR-1a*. Reverse transcription quantitative polymerase chain reaction (RT-qPCR) was used to examine the mRNA levels of *HMGCR* in different tissues; *HMGCR-1a* was the most highly expressed variant in most tissues, with the exception of the skin, esophagus, and uterine cervix, in which *HMGCR-1b* was the most highly expressed transcript. Atorvastatin treatment of HepG2 cells resulted in increased *HMGCR-1b* mRNA levels, but unaltered proximal promoter activity compared to untreated cells. In contrast, *HMGCR-1c* showed a more restricted transcription pattern, but was also induced by atorvastatin treatment.

**Conclusions:**

The gene encoding HMGCR uses alternative, mutually exclusive exon 1 sequences. This contributes to an increased complexity of *HMGCR* transcripts. Further studies are needed to investigate whether *HMGCR* splice variants identified in this study are physiologically functional.

## Background

Human 3-hydroxy-3-methylglutaryl-coenzyme A (HMG-CoA) reductase (HMGCR) is the rate-limiting enzyme in the isoprenoid/cholesterol biosynthesis pathway. The enzyme converts HMG-CoA to mevalonate and is the main target of commonly used lipid-lowering drugs (i.e., statins) [1]. Some of the major intermediates in the pathway are used to synthesise ubiquinone, dolichol, heme A and isoprenoid lipids [2]. The end-product, cholesterol, is the precursor for the synthesis of crucial compounds, such as steroid hormones, bile acid and vitamin D.

HMGCR is encoded by a single gene on chromosome 5 (5q13.3-q14). Two transcript variants are annotated in the National Center for Biotechnology Information (NCBI) Reference Sequence (RefSeq) database; the canonical full-length transcript composed of 20 exons (NM_000859.2) and an alternatively spliced variant lacking exon 13 (NM_001130996.1). Additionally, exon junction microarray experiments suggest the presence of at least three more *HMGCR* splice variants lacking exon 8 (AY429542), exon 18 (AY429543) and exons 17 and 18 (AY429544) [3].

Expression of HMGCR is strictly regulated at the transcriptional and post-transcriptional level. Cholesterol homeostasis is maintained by *HMGCR* transcriptional inhibition and enzyme modifications via negative feedback mechanisms (reviewed in [4]). Under sterol-depleted conditions, such as statin treatment, *HMGCR* transcription is co-ordinately activated with transcription of the low density lipoprotein receptor and other genes in the cholesterol biosynthetic pathway [5].

Genomic diversity is greatly expanded by the process of alternative splicing, by which introns are removed and exons are ligated together to form various mature mRNA transcripts. Alternative splicing has been suggested as another mechanism underlying HMGCR regulation and cholesterol homeostasis [6]. An HMGCR splice variant lacking exon 13 (denoted here as HMGCRΔexon13) was reported to have decreased enzymatic activity and to be less sensitive to statin inhibition [3,7,8]. Exon 13 encodes parts of the catalytic domain and the statin binding site of HMGCR [9,10]. Recently, the major *A* allele of a single-nucleotide polymorphism (SNP, rs3846662, *A* > *G*) located within intron 13 of *HMGCR* was found to be associated with skipping of exon 13 [7]*.* The relative abundance of *HMGCRΔexon13* mRNA has also been reported to be associated with interindividual variation in statin-mediated lipid-lowering responses [8,11]. In the present study, nucleotide sequence database queries identified several novel *HMGCR* splice variants. One of these, *HMGCR-1b*, shows alternative exon 1 usage, with a novel HMGCR N-terminus predicted. We report the mRNA levels of novel *HMGCR* splice variants in a variety of healthy human tissues. Furthermore, we present the regulatory responses of these variants in HepG2 cells upon statin treatment.

## Methods

### Bioinformatic analysis

Inspection of displayed mRNA, expressed sequence tag (EST) and RNA sequencing (RNA-seq) tracks at the *HMGCR* gene locus was performed using the Genome Browser Database provided by the University of California, Santa Cruz (UCSC) on human genome assembly GRCh37/hg19 [12]. The other bioinformatic tools used were Primer3 software [13], Finch TV1.4.0 (Geospiza, Seattle, USA), DNASTAR Lasergene 7.2 (Madison, WI, USA) and the NCBI database of SNPs.

### Cell culture and statin treatment

The HepG2 cell line (American Type Culture Collection, Manassas, VA, USA) was grown in modified Eagle’s minimal essential medium (MEM; ATCC) supplemented with 10% heat inactivated fetal bovine serum (FBS) and and 1× penicillin-streptomycin-glutamine (P/S/G) mixture (Sigma-Aldrich, St Louis, MO, USA) in collagen I-coated tissue culture flasks (BD Biosciences, San Jose, CA, USA). Cells were seeded at 2 × 10^5^ cells/mL in 12-well collagen I-coated plates (BD Biosciences), and the statin treatment was carried out in triplicate on the following day. HepG2 cells were cultivated for 24 hrs in MEM with 1× P/S/G containing 3 mg/mL of human lipoprotein deficient serum (LPDS; Millipore, Billerica, MA, USA), with or without 10 μM of (3 S, 5 S)-Atorvastatin Sodium Salt (Toronto Research Chemicals North York, Ontario, Canada) dissolved in water in a total volume of 1 mL. Two lymphoblastoid cell lines (GM17752 and GM20518; Coriell Institute for Medical Research, Camden, NJ, USA) were cultured according to the manufacturer’s instructions. Sequencing confirmed the *CC* and *AA* genotypes of the rs3761740 polymorphism in GM17752 and GM20518 cell lines, respectively. Statin treatment was carried out as described above, except that 5 × 10^5^ cells/mL of each cell line were seeded into 24-well plates and treated the same day. Cell cultures were maintained at 37°C/5% CO_2_.

### Purification of total RNA from cells

Total RNA from HepG2 cells was isolated using an RNeasy Mini Kit (Qiagen, Venlo, The Netherlands) by following the protocol provided by the manufacturer. DNase digestion with an RNase-Free DNase Set (Qiagen) was used to ensure DNA-free RNA samples. The RNA was eluted in 30 μL of RNase/DNase-free water and stored at −80°C until required. RNA samples were quantified using a NanoDrop ND-1000 spectrophotometer (NanoDrop Technologies, Wilmington, DE, USA). RNA quality was assessed by microfluidic capillary electrophoresis using an Agilent 2100 Bioanalyzer and the RNA 6000 Nano Chip kit (Agilent Technologies, Santa Clara, CA, USA). RNA samples were denatured for 2 min at 70°C prior to cDNA synthesis.

### Total RNA from human tissues

High-quality pooled RNA samples from the Human Total Master panel II and the Human Adult Normal Tissue panel were purchased from Clontech (Mountain View, CA, USA) and BioCat GmbH (Heidelberg, Germany), respectively. Ten random RNA samples were assessed for concentration and integrity as described above. All samples were from anonymous donors and commercially available.

### Synthesis of cDNA

The levels of mRNA expression were analysed using total RNA from tissues (1000 ng) and cells (200 ng) in a 20 μL cDNA synthesis reaction using an Omniscript RT Kit (Qiagen) and a mixture of 2.5 μM oligo-dT and 6.25 μM random hexamers (Applied Biosystems, Foster City, CA, USA). Reverse transcription (RT) was performed at 37°C for 60 min, followed by 95°C for 5 min. The cDNA samples were diluted 1:5 in RNase/DNase-free water and stored at −20°C prior to use as templates for PCR amplification. A pooled cDNA sample was obtained by mixing an aliquot of each of the 44 undiluted cDNA samples and a 1:10 dilution in RNase/DNase-free water.

### PCR

Polymerase chain reaction (PCR) amplification of the different first exons of *HMGCR* in a cDNA pool derived from 44 human tissues (as described above) was performed with HotStarTaq DNA Polymerase (Qiagen) following the manufacturer’s instructions Forward primers (exon1a, 5′-GAG CGT GCG TAA GGT GAG G-3′; exon1b, 5′-GAG AGC AGA AGG AAC GCA CA-3′; exon1c, 5′-GAC ATG GTC CTG CAG AGT CG-3′; and exon1d, 5′-GCA GCA TTG CAT AAA TAC TGT CA-3′) were used in combination with a reverse primer in exon 3 (exon3, 5′-TGT CAG AAT TAT AAT GTC ACT GCT CAA-3′). The cycling conditions were 15 min at 95°C, followed by 37 cycles of 30 s at 94°C, 30 s at 55°C, 1 min at 72°C and a final extension step at 72°C for 10 min. PCR products were separated on 1–3% (w/v) agarose gels. Images were captured using a Kodak Image Station 440CF and visualised using Kodak Molecular Imaging Software, version 4.5 (Eastman Kodak Co., New Haven, CT, USA). The PCR products of interest were excised and purified using the QIAquick Gel Extraction Kit (Qiagen) and confirmed by sequencing.

### Molecular cloning

The full-length *HMGCR-1b* transcript was PCR amplified using a ProofStart DNA polymerase kit (Qiagen) and a cDNA template synthesised from skin (BioCat GmbH). The PCR mix contained 1× proof start buffer, 1× Q-solution, 0.25 U ProofStart, 5 U HotStarTaq plus DNA polymerase, 350 mM dNTP mix, 2 μL of cDNA and 0.4 μM forward primer (exon1b, 5′-CGC AGG AGA GGC ACA TTT CAG-3′) and reverse primer (exon20, 5′-GGG CTG TCT TCT TGG TGC AAG C-3′) in a total volume of 50 μL. The cycling conditions were 5 min at 95°C, followed by 37 cycles of 30 s at 94°C, 1 min at 55°C, 3 min at 72°C and a final extension step at 72°C for 30 min. Purified PCR products were cloned using the CT-GFP Fusion TOPO TA Expression Kit (Invitrogen Corporation, Carlsbad, CA, USA) according to the manufacturer’s instructions. Positive clones were confirmed by sequencing.

### Quantitative PCR (qPCR)

All qPCRs were performed on an ABI 7900HT Real-Time PCR System in a standard 96-well format. TaqMan hydrolysis probes (Custom TaqMan Gene Expression Assay from Applied Biosystems) were designed for specific gene quantification of *HMGCR-1a*, *HMGCR-1b* and *HMGCR-1c*. The following TaqMan primer and probe sets were used: 5′-CCA TGC ATT CGA AAA AGT CTT GAC A-3′ (forward primer), 5′-GGT TCG GTG GCC TCT AGT G-3′ (reverse primer) and the FAM-reporter probe 5′-TCC TTG GAT CCT CCA GAT CT-3′ for targeting the *HMGCR* exon 1a-exon 2 boundary; 5′-AAT GGA TGT CGC ACA CAA GAG A-3′ (forward primer), 5′-CCA TGC ATT CGA AAA AGT CTT GAC A-3′ (reverse primer), and the FAM-reporter probe 5′-CTT GGA TCC TGC GTC TCT-3′ for targeting the *HMGCR* exon 1b-exon 2 boundary; and 5′-TTT CTG GGC TAT ACT AAA TGT GCA TGA-3′ (forward primer), 5′-CCA TGC ATT CGA AAA AGT CTT GAC A-3′ (reverse primer) and the FAM-reporter probe 5′-ACA AGG ATC CAA GGA TTC-3′ for targeting the *HMGCR* exon 1c-exon 2 boundary. Each qPCR mix comprised 10 μL of TaqMan Universal PCR Master Mix, 7 μL of RNase-free water, 1 μL of TaqMan hydrolysis probe and 2 μL of cDNA. The cycling steps were 10 min at 95°C followed by 40 cycles of 95°C for 15 s and 60°C for 1 min. Non-template controls were included in each run. All qPCR experiments were performed at least in duplicate. Samples exhibiting more than a 0.5 quantification cycle (Cq) difference between the duplicates were excluded from the relative quantification analysis. Samples showing Cq > 35 or undetermined were set to Cq = 38 and were considered not detectable.

### Reference gene selection

The stability of 12 reference genes from Applied Biosystems (see [14] for assay IDs) was checked to confirm the reliability of gene expression results across the tissue samples tested. Five *(GAPDH, PGK1, SDHA, CTBP1, GOLGA1)* were found by geNorm [15,16] to give the most suitable normalisation factors for quantification of relative expression levels. For the atorvastatin treatment experiments in HepG2 cells, the *PPIA, TBP, PGK1, ACTB, GAPDH, UBC, PPIB,* and *SDHA* reference genes were tested. Two reference genes, *PPIA* and *TBP*, were found by geNorm to be the most stable and therefore were suitable for normalisation. The mean expression value was used for the normalisation, according to the “ΔΔCq method” for comparing relative expression results between treatments [17]:

(1) $$\Delta \Delta \text{Cq}=2^{-(\Delta \text{Cqtreated }-\Delta \text{Cq non}-\text{treated})}$$

### Reporter vector construction

Different regions of the human *HMGCR* promoter covering −1626 to +12 (relative to the annotated *HMGCR* transcription start) were amplified by PCR. Genomic DNA from the Human Variation Panels (NA07055; Coriell Institute for Medical Research) heterozygous for the HMGCR promoter SNP *C > A* (rs3761740) were used as PCR templates. The PCR primers were: 5′-AGC GGT ACC GCC ATT TAC ACT AAT GGG TAA AT-3′ (forward primer) and 5′-AGT AAG CTT GAC CAA TAA GAG AGG ATC GTT CG-3′ (reverse primer) for construction of the reporter vector 1 (−559 to +12); 5′-AGC GGT ACC AAT GGG TAG GCA TAT CCA AGG-3′ (forward primer) and 5′-AGT AAG CTT CTG CGT CTT CTG TGC GTT C-3′ (reverse primer) for construction of reporter vectors 2 and 3 (−1009 to −615); and 5′-AGC GGT ACC TTG ACA AGG CTG CTA AGA GAA CA-3′ (forward primer) for construction of reporter vectors 4 and 5 (−1626 to −615) in combination with the same reverse primer used for reporter vectors 2 and 3. Restriction sites for *KpnI* and *HindIII* are underlined in the forward and reverse primer sequences, respectively. The PCR was performed using a Phusion High-Fidelity PCR kit (Finnzymes Oy, Espoo, Finland) according to the manufacturer’s instructions. Cycling conditions were as follows: 30 s at 98°C, followed by 35 cycles of 10 s at 98°C, 30 s at 63°C, 1 min at 72°C and a final extension step of 10 min at 72°C. The PCR-amplified promoter regions were cloned into the *KpnI* and *HindIII* restriction sites of the promoterless firefly luciferase vector pGL.4.10 (Promega, Madison, WI, USA).

### Transfection and reporter gene assay

HepG2 cells were seeded on the day prior to transfection at 1.25 × 10^5^ cells/mL into a 24-well collagen I-coated plate (BD Biosciences) in 0.5 mL of culture medium. For each transfection, 485 ng of the firefly luciferase empty control vector (pGL4.10) or a test reporter vector was co-transfected with 15 ng of renilla luciferase internal control vector (pGL4.73) to normalise for transfection efficiency. Transfection was carried out in triplicate using 1.25 μL of FuGENE HD (Roche Diagnostics Corporation, Indianapolis, IN, USA) reagent per well. Cells were washed with 1 mL 1× phosphate-buffered saline at 24 h post-transfection and then treated with 100 μL of Passive Lysate Buffer supplied in the Dual-Glo luciferase assay kit (Promega). After gentle rocking (15 min), the lysates were assayed for luciferase activity with the Dual-Glo luciferase assay system according to the manufacturer’s instructions. Firefly luciferase and *renilla* luciferase activities were measured using the Wallac 1420 Victor™ luminometer in a 96-well Wallac B&W Isoplate™ (Perkin Elmer, Turku, Finland). Delay and measurement times were set to 2 s and 10 s, respectively. The linear range of light detection was determined using a dilution series of QuantiLum Recombinant Luciferase (Promega). Firefly luciferase activity was normalised to *renilla* luciferase expression. Luciferase (Luc) activity induction was calculated as fold-change according to the following equation:

(2) $$\text{Fold}-\text{change }=\left[\text{Mean }\left(\text{Firefly}/\text{Renilla}\right)\text{test reporter vector}\right]/\left[\text{Mean }\left(\text{Firefly}/\text{Renilla}\right)\text{emptycontrol vector}\right]$$

## Results

### Identification of *HMGCR* splice variants

**Figure 1:**
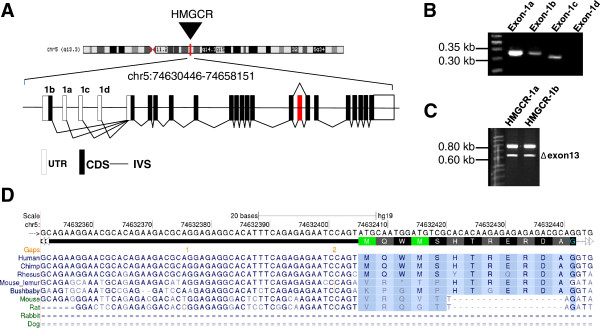
**Alternative splicing of *HMGCR.*** (**A**) Genomic organisation of human *HMGCR* at chromosome 5q13.3. Exons are separated by introns (IVS) and represented by white and black boxes illustrating predicted untranslated regions (UTR) and coding sequences (CDS), respectively. The canonical exon 1a is part of the *HMGCR* reference sequence (NM_000859) and exons 1b, 1c and 1d are predicted from published ESTs. The red box indicates the cassette exon 13, which is skipped in an alternative *HMGCR* transcript (NM_001130996). (**B**) Validation of exon 1a-, 1b-, 1c- and 1d-transcription by RT-PCR using exon-specific primers and a cDNA pool of 44 tissues visualised on a 3% (w/v) agarose gel. RT-PCR identified *HMGCR* transcripts with different first exons. Transcription of exon 1d could not be confirmed. (**C**) Exon 13 (159 bp) skipping (Δexon13) of both *HMGCR-1a* and *HMGCR-1b* identified by PCR and visualised on a 2% (w/v) agarose gel. cDNA synthesised from skin was used to amplify the *HMGCR* transcripts from exons 1a and 1b, respectively, through exon 16 followed by nested PCR from exon 10 through exon 14. The existence of *HMGCR-1b* Δexon13 was confirmed by cloning and sequencing. (**D**) Multiple sequence alignments of *HMGCR* exon 1b in different mammalian species showing conservation of exon 1b only in higher primates. The alignment was created using the UCSC Genome Browser “Vertebrate Multiz Alignment & Conservation (46 Species)” track. An in-frame start codon (ATG) is preserved in higher primates and presented as the methionine single letter code (M). Single dashes indicate alignment gaps, and double dashes represent one or more unalignable bases in the gap region.

Bioinformatic analysis and visual inspection of the region between *HMGCR* exon 2 and its adjacent, 5′-flanking gene in the UCSC genome browser indicated at least three alternative first exons, each spliced to the constitutive exon 2 (Acc. No: AK296499, DA809145, BX952828, designated exons 1b, 1c and 1d, respectively; Figure 1A). Tracks of published RNA-seq data indicated transcription of exon 1b, whereas no convincing evidence was found for transcription of exons 1c and 1d. Transcription of exons 1b and 1c was confirmed by RT-PCR (Figure 1B) of the cDNA pool derived from 44 different tissues. Expression of the predicted exon 1d could not be confirmed by RT-PCR in any of the investigated tissues. The open reading frame of the canonical *HMGCR* transcript (=888 amino acids) is maintained in exon 1c, but sequence analysis of exon 1b predicted an additional 20 amino acids at the N-terminus. We cloned and sequenced *HMGCR-1b* (exon 1b– exon 20) and confirmed that exons 1a and 1b were mutually exclusive. We also found that *HMGCR-1b* transcripts were subject to exon 13 skipping (*HMGCR-1bΔ13*) (Figure 1C). Exon 1b is potentially translated in certain primates as indicated by alignment of nucleotide sequences from different vertebrate species (Figure 1D).

### Tissue distribution of *HMGCR-1a*, *-1b* and *-1c*

**Figure 2:**
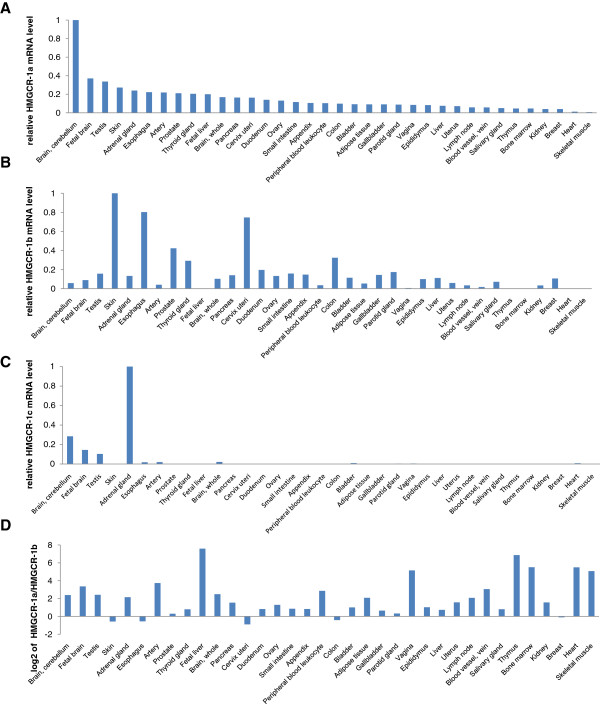
**Expression of *HMGCR-1a *,*-1b *and *-1c *in various human tissues. ***HMGCR-1a*, *-1b* and *-1c* mRNA levels were determined by RT-qPCR in various human tissues using exon 1-specific primers. (**A**) *HMGCR-1a*, (**B**) *HMGCR-1b* (**C**) *HMGCR-1c* mRNA levels were normalised to a tissue-dependent factor based on five reference genes and calculated as ratios relative to the highest mRNA level (reference), which was set to 1. (**D**) Ratios of *HMGCR-1a* and *HMGCR-1b* mRNA levels are given on a logarithmic scale (base 2). *HMGCR-1b* is in low abundance compared with the canonical *HMGCR-1a* in most of the analysed tissues (ratio >0), except in the skin, esophagus, uterine cervix and colon (ratio <0).

The RT-qPCR experiments showed variable levels of alternative first exons of *HMGCR* in 36 different tissues (Figure 2). The canonical *HMGCR-1a* transcript was ubiquitously expressed at the highest levels in the cerebellum, fetal brain, testis, skin and adrenal gland (Figure 2A). The highest expression of *HMGCR-1b* was detected in the skin, esophagus, uterine cervix and prostate (Figure 2B). *HMGCR-1b* mRNA levels were below detection levels (Cq > 35.0) in six tissues. The level of *HMGCR-1a* in liver (pooled from three different donors) was approximately two-fold higher than that of *HMGCR-1b*. We observed higher *HMGCR-1a* expression in most of the tissues analysed with the exception of the skin, esophagus, uterine cervix and colon, in which the levels of *HMGCR-1b* were higher (approximately 1.5–2.0-fold; Figure 2D). *HMGCR-1c* was detectable (Cq < 35) in only nine of the tissues analysed and was less abundant than *HMGCR-1a.* The highest expression of *HMGCR-1c* was detected in the cerebellum, fetal brain, testis and adrenal gland (Figure 1C). *HMGCR-1c* could not be quantified in the skin or uterine cervix (Cq > 35), where *HMGCR-1b* was relatively abundant.

### Expression analysis before and after atorvastatin treatment in HepG2 cells

**Figure 3:**
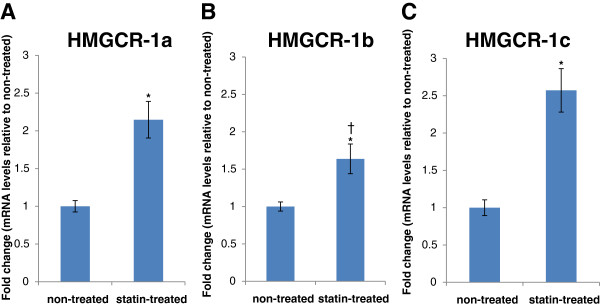
**Upregulation of *HMGCR *transcript variants in HepG2 cells treated with atorvastatin.** HepG2 cells were incubated in culture medium with lipoprotein deficient serum (LPDS; non-treated) and 10 μM atorvastatin + LPDS for 24 h (treated). The mRNA levels of (**A**) *HMGCR-1a* (**B**) *HMGCR-1b* and (**C**) *HMGCR-1c* were measured by RT-qPCR using exon1-specific primers and a set of validated reference genes for normalisation. The results are shown as the mean fold-change compared with mRNA levels in non-treated cells. Error bars indicate standard errors (± S.E), n = 5. *Statistically significant differences between non-treated and treated (*p* < 0.05) were determined using Student’s *t*-test. †The magnitude of statin-induced upregulation of *HMGCR-1b* was significantly lower (*p* < 0.05) than that of *HMGCR-1a* and *HMGCR-1c*, respectively, as determined by a paired-sample Student’s *t*-test.

HepG2 cells, a model system for cholesterol biosynthesis studies, were treated with atorvastatin for 24 h. The RT-qPCR experiments showed significant mRNA induction (*p* < 0.05) for *HMGCR-1a* (2.1 ± 0.2-fold (mean ± SE; Figure 3A), *HMGCR-1b* (1.6 ± 0.2-fold; Figure 3B) and *HMGCR-1c* (2.6 ± 0.3-fold; Figure 3C) in cells after atorvastatin treatment compared with untreated cells. The magnitude of induction was significantly different between *HMGCR-1a* and *HMGCR-1b,* and between *HMGCR-1b* and *HMGCR-1c* (*p* < 0.05), but not between *HMGCR-1a* and *HMGCR-1c* (*p* = 0.16). The *HMGCR-1c* and *HMGCR-1b* splice variants were less abundant (approximately 60-fold lower) in HepG2 cells compared with the canonical *HMGCR-1a*. The fact that exon 1b was found to be approximately two-fold lower than exon 1a in the liver suggests that there are major differences in the expression levels of these transcripts, either between different liver donors or because of cell immortalisation and/or an artificial cell culture environment for the HepG2 cells.

### *HMGCR* promoter analysis

**Figure 4:**
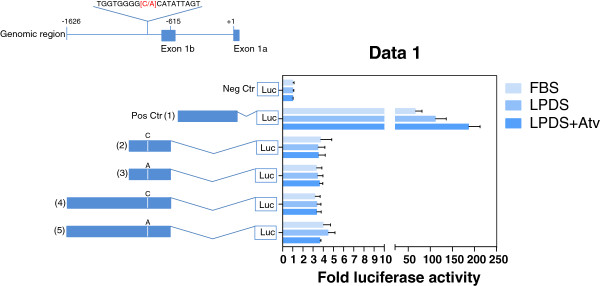
**Promoter activities of the canonical *HMGCR-1a *and the putative *HMGCR-1b *promoter in HepG2 cells.** Solid bars illustrate different sizes of genomic sequences upstream of exon 1a and exon 1b that were cloned into pGL4.10. The *HMGCR* promoter SNP (*C > A*, rs3761740) is highlighted in red and represented by both alleles. Luciferase activity was measured in the lysates of transfected cells incubated for 24 h in fetal bovine serum (FBS), lipoprotein-deficient serum (LPDS) or LPDS with 10 μM atorvastatin (Atv). The promoter activity is shown as the mean fold-change in firefly luciferase activity relative to the mean firefly luciferase activity of the empty pGL4.10 vector, which was set to 1 after correction for transfection efficiency using *Renilla* luciferase activity. The results are the mean of three independent experiments performed in triplicate. Error bars indicate standard errors (± S.E).

Bioinformatics showed that exon 1b is located 35 bp upstream of the CpG island in the previously characterised *HMGCR* promoter. We performed luciferase reporter gene assays in HepG2 cells under various conditions to investigate the promoter activity driving the transcription of *HMGCR-1b* and the genetic influence of the promoter SNP rs3761740, which is close to exon 1b. The promoter region upstream of exon 1a (reporter vector 1, which was used as a positive control) was active under standard culture conditions with 10% FBS (Figure 4). The exon 1a promoter was further activated under sterol-depleted conditions with LPDS (1.7 ± 0.2-fold) and LPDS + atorvastatin (3.0 ± 0.6-fold) when compared with the activity of transfected cells cultured under standard conditions, containing 10% FBS. The reporter vectors 2, 3, 4 and 5, each containing genomic fragments upstream of exon 1b, exhibited low promoter activities that were not induced by sterol depletion (LPDS), or by higher degrees of sterol depletion (LPDS + atorvastatin). The minor *A* allele of the promoter SNP *C > A* (rs3761740) did not affect the promoter activity of exon 1b when compared with the major allele *C* reporter vector. The effect of the promoter SNP *C > A* (rs3761740) on *HMGCR* exons 1a and 1b mRNA levels was investigated after atorvastatin treatment in two different lymphoblastoid cell lines homozygous for either allele. The RT-qPCR experiments (n = 5) showed similar induction of *HMGCR-1b* mRNA levels after atorvastatin treatment in both lymphoblastoid cell lines, independent of the allelic status. *HMGCR-1b* was induced 1.2 ± 0.1-fold (mean ± SE) and 1.3 ± 0.1-fold in cells with the major *CC* and the minor *AA* genotype of rs3761740, respectively. The magnitude of induction was not significantly different between the two different cell lines. The canonical *HMGCR-1a* transcript was upregulated 1.7 ± 0.1-fold in both lymphoblastoid cell lines after atorvastatin treatment.

## Discussion

HMGCR is the key enzyme in the cholesterol biosynthesis pathway and is the major target of cholesterol-lowering statin drugs. Here, we demonstrate the existence of alternative exon 1 usage in human *HMGCR*, resulting in several novel transcript variants. One of these, *HMGCR-1b*, encodes a novel N-terminus which potentially extends the membrane domain by 20 amino acids compared with the canonical sequence. The canonical HMGCR protein is composed of 888 amino acids with a regulatory, sterol-sensing transmembrane domain (residues 10 – 339), a flexible linker (residues 340 – 449) and a C-terminal catalytic region (residues 450 – 888) [18]. We studied *HMGCR-1b* at the genomic and transcriptional levels. Whether the translation machinery actually recognises the first AUG as a start codon to produce a protein with 20 additional amino acids in the membrane domain remains to be determined. Unfortunately, initial experiments using rabbit antibodies raised against the novel N-terminus of the predicted HMGCR-1b polypeptide exhibited non-specific binding (data not shown).

In mammalian genomes, only one gene encodes the HMGCR enzyme, which mainly resides in the membrane of the endoplasmic reticulum [19,20]. A minor fraction (approximately 5%) of the reductase activity has been demonstrated in rat liver [21-23] and mouse brain [24] to be localised in the matrix of peroxisomes. The signal that targets HMGCR to peroxisomes has not yet been determined. The *HMGCR-1b* transcript identified in this study is remarkably less abundant than the canonical *HMGCR-1a* in most of the tissues analysed, and it is tempting to speculate that exon 1b might encode the missing HMGCR peroxisomal targeting information [25]. However, bioinformatic analysis of the N-terminus of the hypothetical HMGCR-1b protein did not reveal any peroxisomal targeting signals (data not shown).

Our finding that *HMGCR* generates multiple 5′ end transcripts is not unusual in the human genome [26]. There are numerous other examples in the literature. The gene locus encoding the uridine diphosphate–glucuronosyltransferase 1 family contains several different coding first exons, which generate at least nine different transcripts encoding various isozymes with unique N-termini and substrate specificities [12]. Another example is the *CYP19A1* gene encoding aromatase, the rate-limiting enzyme of estrogen biosynthesis, which contains alternative non-coding first exons affecting RNA stability and aromatase levels in tissues. Differential use of non-coding first exons is suggested to be an important mechanism of post-transcriptional regulation of aromatase gene expression [27].

Whether an alternatively spliced variant is functional is considered in the context of tissue-specific expression and conservation in most studies [28]. The mRNA levels for the alternative *HMGCR-1b* splice variant were more abundant than the canonical *HMGCR-1a* levels in epithelial tissues, such as skin. Skin is one of the major sites for cholesterol production in the body [29], where HMGCR activity and cholesterol synthesis are crucial for hindering fluid loss from the body by maintaining the permeability barrier [30]. In addition, the production of non-cholesterol isoprenoid intermediates by HMGCR has been reported to be important during acute wound healing [31]. Sequence alignment of *HMGCR-1b* did not show conservation of the open reading frame in all terrestrial animals. Therefore, it remains to be determined whether the *HMGCR-1b* splice variant exerts an epithelial or skin-specific role in sustaining the epithelial barrier function in certain primates.

Tissue-specific gene expression may be regulated via differential gene promoter activity [32]. The weak promoter activity of the genomic region upstream of exon 1b is in accordance with the rather low, basal mRNA expression level of exon 1b found in HepG2 cells. Unexpectedly, the alternative promoter activity in the reporter assay was not induced by sterol depletion, although there was an increase in the *HMGCR-1b* mRNA level in HepG2 cells. Based on these results, the increased *HMGCR-1b* mRNA level due to sterol depletion in HepG2 cells most likely results from post-transcriptional regulation and/or regulatory promoter DNA sequences not captured in our reporter vector constructs. Moreover, previous studies have identified a functional *C > A* SNP (rs3761740) in the *HMGCR* promoter [23,33]. Keller et al. identified a sterol regulatory element (SRE) in this position. They found that the minor *A* allele was more responsive to the overexpression of sterol regulatory element-binding protein 2, compared with the major *C* allele [33]. In our experiments, this promoter SNP, which is close to the transcription start of exon 1b, did not contribute significantly to the proximal promoter activity or mRNA level of *HMGCR-1b* or to the transcription of the canonical *HMGCR-1a* variant upon sterol depletion.

Approximately half of human genes are predicted to have alternative promoters [34]. A comparative study of different promoter features in human and mouse genes reported a distinct class of “non-conserved” promoters in humans [35]. Alternative promoters differ from the major promoter in their responsiveness to a specific condition, resulting in different expression levels in tissues or developmental stages and thereby increasing the flexibility of gene expression. Given that the putative *HMGCR-1b* promoter identified in our study (i) exhibits a different response to statin treatment compared with the canonical *HMGCR* promoter, (ii) is of minor usage, (iii) is lacking CpG islands, and (iv) that the *HMGCR-1b* transcript shows an expression pattern distinct from that of *HMGCR-1a*, it seems likely that the *HMGCR-1b* promoter represents an alternative, non-conserved *HMGCR* promoter that contributes to the expressional diversity of this gene.

With respect to *HMGCR-1a*, we also found that exon 13, which encodes amino acids in the catalytic region, is a cassette exon in the *HMGCR-1b* transcript, meaning that this exon is either included *(HMGCR-1b)* or skipped *(HMGCR-1bΔ13).* The catalytically active HMGCR is formed as a tetramer with two dimers [10]. Medina et al. have proposed that the inactive HMGCR lacking exon 13 modulates the HMGCR enzyme activity by being part of the catalytically active tetramer and thereby reducing its total enzyme activity [11]. Further studies are needed to clarify whether the delta-exon 13 variant of the *HMGCR-1b* transcript also contributes to HMGCR enzyme activity modulation and statin response.

## Conclusions

We have shown that human *HMGCR* uses different first exons to produce multiple alternatively spliced variants, thereby contributing to increased transcriptional complexity. We have designated these splice variants *HMGCR-1a*, *HMGCR-1b* and *HMGCR-1c*. The novel *HMGCR* exon 1 *(HMGCR-1b)* we identified contains an in-frame ATG translational start codon predicting an extended N-terminus of HMGCR. Moreover, we showed that this transcript variant is differentially expressed in a wide variety of healthy human tissues, and that its mRNA levels are regulated upon statin treatment. Further studies are needed to elucidate potential physiological roles of differential first exon usage in *HMGCR*.

## Abbreviations

HMGCR: 3-Hydroxy-3-Methylglutaryl-Coenzyme A reductase; HMG-CoA: 3-Hydroxy-3-Methylglutaryl-Coenzyme A; SNP: Single-Nucleotide Polymorphism; EST: Expressed Sequence Tag; RNA-seq: RNA sequencing; MEM: Modified Eagle’s Minimal essential medium; FBS: Fetal Bovine Serum; LPDS: Lipoprotein Deficient Serum; RT: Reverse Transcription; PCR: Polymerase Chain Reaction; qPCR: Quantitative Polymerase Chain Reaction; Luc: Luciferase; Atv: Atorvastatin.

## Competing interests

The authors declare that they have no competing interests.

## Authors’ contributions

CS carried out the experimental work and drafted the manuscript with assistance from RMG and MKK. AP, MKK and JPB participated in the study design, interpretation of data and revising the manuscript. All authors read and approved the final manuscript.
